# Undifferentiated autoinflammatory disease in adults: a prospective study in 61 patients

**DOI:** 10.1186/s13023-025-03685-5

**Published:** 2025-04-08

**Authors:** Junke Miao, Di Wu, Min Shen

**Affiliations:** 1 Department of Rare Diseases, Peking Union Medical College Hospital (PUMCH), Chinese Academy of Medical Sciences & Peking Union Medical College; State Key Laboratory of Complex Severe and Rare Diseases, PUMCH, Beijing, 100730 China; 2 Department of Rheumatology and Clinical Immunology, PUMCH; National Clinical Research Center for Dermatologic and Immunologic Diseases (NCRC-DID), Ministry of Science & Technology; Key Laboratory of Rheumatology and Clinical Immunology, Ministry of Education, Beijing, 100730 China

**Keywords:** Systemic autoinflammatory diseases, Undifferentiated autoinflammatory diseases, Whole-exome sequence

## Abstract

**Backgrounds:**

Undifferentiated or undefined systemic autoinflammatory disease (uSAID) encompasses a group of rare, heterogeneous diseases characterized by the features of well-defined systemic autoinflammatory diseases (SAIDs), but lacking diagnostic phenotypes or genetic confirmation. We aimed to describe the phenotypes, genotypes and treatment responses of Chinese adult patients with uSAID.

**Methods:**

The final diagnosis of uSAID was reached in 61 patients, whose organ-specific inflammation was compared and three subgroups were identified based on phenotypic similarities to well-defined SAIDs. Phenotypes, genotypes and treatment responses were analyzed in these subgroups.

**Results:**

Among the 61 uSAID patients, 17 had disease-onset during childhood, and 44 had adult-onset. Compared to those without pulmonary manifestations, patients with pulmonary involvement exhibited higher frequencies of myalgia, skin lesions, cardiac involvement, gastrointestinal involvement, urinary involvement, lymphadenopathy, headache, and intellectual impairments. Twenty-four patients exhibited monogenic SAID-like phenotypes, 12 had polygenic SAID-like phenotypes, and the remaining 25 were categorized as having atypical phenotypes. Among the 53 patients followed, 25% (13/53) improved spontaneously with complete or partial recovery independent of therapy. Patients with atypical phenotypes had the highest spontaneous remission rate (10/23, 43%).

**Conclusion:**

This study is the first to describe the clinical and genetic features of a cohort of Chinese adult patients with uSAID. Patients with pulmonary manifestations may be more prone to developing complex phenotypes, while those with atypical phenotypes have a high rate of spontaneous remission, indicating a favorable prognosis.

**Supplementary Information:**

The online version contains supplementary material available at 10.1186/s13023-025-03685-5.

## Background

Systemic autoinflammatory diseases (SAIDs) are disorders caused by dysregulation of the innate immune system, characterized by recurrent inflammation without pathogenic autoantibodies or antigen-specific T cells [1, 2]. Well-defined SAIDs include monogenic SAIDs caused by loss-of-function or gain-of-function mutations in SAID-associated genes, such as familial Mediterranean fever (FMF), *NLRP3*-associated autoinflammatory disease (*NLRP3*-AID), TNF receptor-associated periodic syndrome (TRAPS), as well as polygenic SAIDs caused by the genetic and environmental factors such as periodic fever, aphthous stomatitis, pharyngitis and adenitis (PFAPA) syndrome, Behçet’s syndrome (BS), inflammatory bowel disease (IBD), chronic nonbacterial osteomyelitis (CNO), adult-onset Still’s disease (AOSD) and systemic juvenile idiopathic arthritis (sJIA) [3]. However, many patients exhibit characteristics of SAIDs but don’t meet any clinical criteria of monogenic or polygenic SAIDs, and their genotypes can’t fully explain their phenotypes, classifying them as undifferentiated or undefined SAID (uSAID) [4–6].

Given that uSAID represents a group of heterogeneous diseases with limited related studies and no well-established diagnostic criteria, distinguishing these disorders from other conditions such as infections and connective tissue diseases is challenging. Furthermore, treatment of these patients presents a significant challenge for physicians. In this study, we reported the first cohort of Chinese adult patients with uSAID, summarizing their phenotypes, genotypes and prognosis to share our clinical experience and contribute to further exploration of uSAID.

## Materials and methods

### Patients

From April 2015 to April 2021, 274 adult patients (aged ≥ 16 years old) suspected of having SAIDs according to the 2010 definition [2] were enrolled at the Department of Rheumatology, Peking Union Medical College Hospital. Inclusion criteria were: (1) recurrent inflammatory signs and symptoms such as recurrent fever, arthralgia/arthritis, dermatitis, ocular manifestations and headache, with symptom-free intervals; (2) elevation of acute phase reactants including [C-reactive protein (CRP), erythrocyte sedimentation rate (ESR) and pro-inflammatory cytokines such as interleukin (IL)-6, tumor necrosis factor (TNF)-α and IL-8] during attack episodes, normalizing between episodes; (3) lack of response to antibiotics. Microbiological testing such as blood cultures, viral polymerase chain reaction (PCR) panels and serological tests for common pathogens and tests of autoantibodies were also performed and patients diagnosed as malignancies, infectious diseases, autoimmune diseases, or other conditions explaining their recurrent inflammation were excluded.

This prospective observational study was approved by the Institutional Review Board of Peking Union Medical College Hospital and conducted in accordance with the Declaration of Helsinki. Informed consents were obtained from all participants.

### Diagnostic criteria

The diagnosis of SAIDs in this study was based primarily on the clinical criteria and, to a lesser extent, on the genetic analyses [7–12]. Whole exome sequencing (WES) using next-generation sequencing (NGS) was performed on each patient, except for those who could be diagnosed with polygenic SAIDs based on typical clinical manifestations. For patients who met the clinical criteria for monogenic SAIDs as proposed by Gattorno et al., but whose WES results did not support the clinical diagnosis, Sanger sequencing was conducted to identify mutations in the suspected genes [13]. Additional investigations, such as circulating cytokine levels, were performed as per clinical needs. Interferon (IFN) signature analysis was conducted for patients suspected of having interferonopathies. Patients were treated and followed up at our center every 3 to 6 months. Unlike other studies, physicians in this study re-evaluated patients by considering their symptom diaries and responses to therapies for trials during follow-up, especially for those resembling FMF or PFAPA. Reanalysis of the WES results were also conducted several years later to account for newly defined SAIDs. If no definite diagnosis was reached, the patient was classified as having uSAID and enrolled in our study.

### Phenotypes and genotypes

Demographic information and clinical data, including clinical manifestations and treatment responses at diagnosis and follow-up, were prospectively collected. To assess constitutional and organ-specific inflammation in uSAID patients, we compared the occurrence of different symptoms in relation to organ involvements.

Regarding NGS results, exonic and nonsynonymous mutations with a minor allele frequency (MAF) < 0.03 in SAID-associated genes (Supplementary Table S1) were selected after filtering benign and likely benign variants based on the Infevers database (https://infevers.umai-montpellier.fr/web/), ClinVar database and American College of Medical Genetics guidelines [14]. Variants not previously reported were termed “novel variants.” Some single nucleotide polymorphisms (SNPs), such as *NLRP12* F402L, which we had previously published, were retained due to their potential significance in Chinese adult patients with SAIDs [15]. We adjusted the heterozygosity cut-off frequency to 10–90% for SAIDs-associated genes during bioinformatic analysis to detect possible somatic mosaicism.

### Subgroups of uSAID patients

Considering the significant variability among uSAID patients and the limited utility of WES results, patients were classified into three subgroups based on whether their phenotypes resembled any well-defined SAIDs to explore their prognosis further. The “monogenic SAID-like” group included patients suspected of having monogenic SAIDs based on their phenotypes, despite no pathogenic variants being detected. The “polygenic SAID-like” group comprised patients whose phenotypes resembled polygenic SAIDs but who did not fully meet the diagnostic criteria. Those with typical autoinflammatory symptoms but whose clinical presentations did not match any classic SAIDs were classified as the “atypical” group.

### Statistics analysis

Continuous variables were expressed as medians and ranges and were assessed using the independent samples t-test or rank-sum analysis. Categorical variables were described as frequency and compared using the Chi-square test or Fisher’s exact test. All statistical tests were two-sided, and the significance level of *p* was set as 0.05. Analyses were performed using IBM SPSS Statistics (Version 26).

## Results

### Demographic data and clinical features

**Fig. 1:**
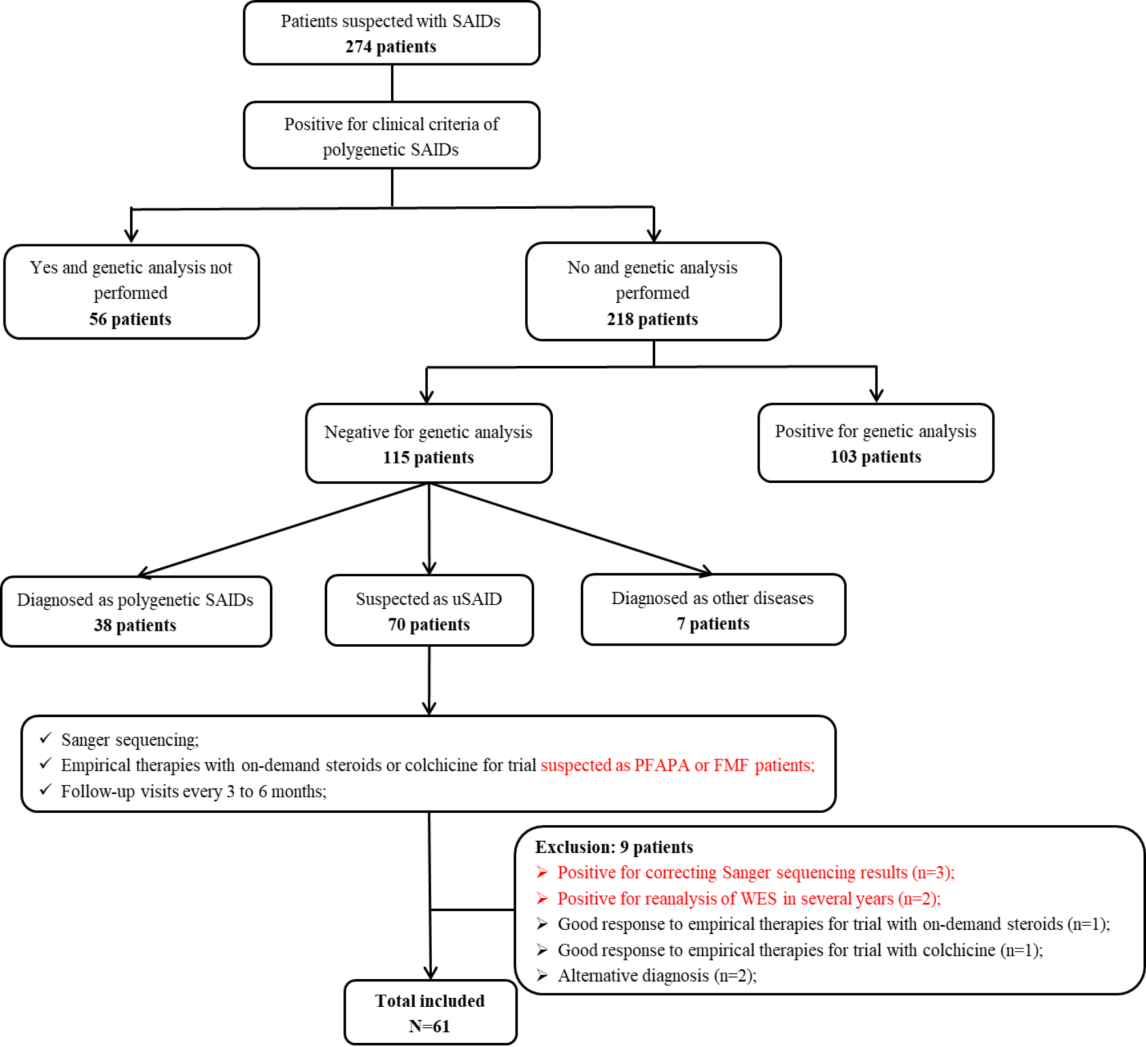
Flow chart for the study cohort

Among the 274 patients suspected of SAIDs, 103 were diagnosed with definite SAIDs based on WES, and 45 were identified as having polygenic SAIDs, such as CNO, AOSD, and PFAPA syndrome, or other connective tissue diseases, after excluding monogenic SAIDs through WES. Of the remaining 70 patients with presumed uSAID, 3 were later diagnosed as monogenic SAIDs including FMF and *NLRP12*-AID due to the detection of variants in suspected genes with high MAF by re-performing Sanger sequencing. Another 2 patients were diagnosed with deficiency of adenosine deaminase 2 (DADA2) and pyogenic arthritis, pyoderma gangrenosum and acne (PAPA) through reanalysis of WES several years later. Empirical therapies using on-demand steroids or colchicine were administered to patients suspected of having PFAPA or FMF for trial purposes, with two patients responded well and subsequently being excluded from our cohort. During follow-up, two additional patients were classified as having rheumatoid arthritis (RA) and CNO based on clinical manifestations and the bone scans results. Ultimately, 61 patients were diagnosed with uSAID and enrolled in our study (Fig. 1).

All 61 patients were Chinese, with a median disease onset age of 26 years (range 16–37 years) (Table 1). Seventeen patients (28%) had disease onset during childhood, while 44 (72%) were adult-onset. The male-to-female ratio was 36:25. Eleven patients (18%) reported a family history of recurrent fever or similar inflammatory symptoms. Among the 61 uSAID patients, constitutional manifestations were common, with periodic fever (95%) and fatigue (67%) being the most prevalent. Other frequent manifestations included gastrointestinal (70%), articular-skeletal (61%) and neurological (52%) involvement. The frequency of skin lesions (71% vs. 39%, *p* = 0.025), impaired vision (24% vs. 5%, *p* = 0.046) and chest pain (24% vs. 5%, *p* = 0.046) were significantly higher in patients with childhood-onset compared to those with adult-onset (Table 1).

**Table 1:** Summary of the demographic data and clinical manifestations of child-onset and adult-onset patients with uSAID

	Child-onset patients *n* = 17	Adult-onset patients *n* = 44	All patients *n* = 61
Demographic data			
Ratio of gender (M: F)	10:7	13:9	36:25
Age at disease onset, median (years), range (years)	9 (0–15)	32 (16–63)	25 (0–63)
Age at diagnosis, median (years), range (years)	26 (16–37)	37 (18–64)	34 (16–64)
Delayed diagnosis, median (years), range (years)	16 (1–31)	5 (1–22)	8 (1–31)
Family history, *n* (%)	5 (29)	6 (14)	11 (18)
Clinical manifestations, *n* (%)			
Constitutional symptoms	17 (100)	43 (98)	60 (98)
Weight loss	5 (28)	13 (30)	18 (29)
Fever	17 (100)	41 (93)	58 (95)
Cold-induced	1 (6)	8 (18)	9 (15)
Fatigue	12 (71)	29 (66)	41 (67)
Myalgia	6 (35)	22 (50)	28 (46)
Articular-skeletal involvement	12 (71)	25 (57)	37 (61)
Arthralgia/arthritis	11 (65)	25 (57)	36 (59)
Dermatological involvement^*^	12 (71)	17 (39)	29 (48)
Cutaneous rash^*^	12 (71)	17 (39)	29 (48)
Erythema nodosa	5 (29)	7 (16)	12 (20)
Mucocutaneous involvement	9 (53)	14 (32)	23 (38)
Oral ulcers	8 (47)	10 (23)	18 (30)
Dry mouth	2 (12)	11 (25)	13 (21)
Ocular involvement	7 (41)	16 (36)	23 (38)
Periorbital oedema	0 (0)	3 (7)	3 (5)
Dry eyes	1 (6)	6 (14)	7 (11)
Conjunctivitis	3 (18)	7 (16)	10 (16)
Impaired vision^*^	4 (24)	2 (5)	6 (10)
Other ocular symptoms^$^	5 (29)	5 (11)	10 (16)
Otolaryngological involvement	7 (41)	21 (48)	28 (46)
Sensorineural deafness	2 (12)	3 (7)	5 (8)
Tinnitus	3 (18)	2 (5)	5 (8)
Pharyngitis/ Tonsillitis	5 (29)	16 (36)	21 (34)
Cardiac involvement	3 (18)	4 (9)	7 (11)
Effusion/ Pericarditis	1 (6)	3 (7)	4 (7)
Gastrointestinal involvement	11 (65)	32 (73)	43 (70)
Abdominal pain/diarrhea	5 (29)	15 (34)	20 (33)
Nausea/vomiting	4 (24)	14 (32)	18 (30)
Splenomegaly	7 (41)	13 (30)	20 (33)
Other gastrointestinal manifestations^#^	7 (41)	16 (36)	23 (38)
Pulmonary involvement	7 (41)	13 (30)	20 (33)
Effusion/ Pleuritis	2 (12)	4 (9)	6 (10)
Interstitial lung disease	2 (12)	1 (2)	3 (5)
Chest pain^*^	4 (24)	2 (5)	6 (10)
Urinary involvement	2 (12)	6 (14)	8 (13)
Proteinuria	2 (12)	4 (9)	6 (10)
Haematuria	1 (6)	2 (5)	3 (6)
Neurological involvement	6 (35)	26 (59)	32 (52)
Headache	6 (35)	25 (57)	31 (51)
Intellectual impairments	1 (6)	2 (5)	3 (5)
Other neurological manifestations^£^	3 (18)	5 (11)	8 (13)
Lymphadenopathy	6 (35)	21 (48)	27 (44)

^*^ The frequencies of symptoms with statistically significant difference between child-onset and adult-onset patients (*p-*values < 0.05);

^$^ Other ocular manifestations included keratitis, scleritis, uveitis and optic atrophy;

^#^ Other gastrointestinal manifestations included ulcers, hemorrhage, hepatomegaly, abnormal liver function and intestinal obstruction;

^£^ Other neurological manifestations include dizziness, epileptic seizure, intracranial calcification, relapsing meningitis and encephalatrophy

**Fig. 2:**
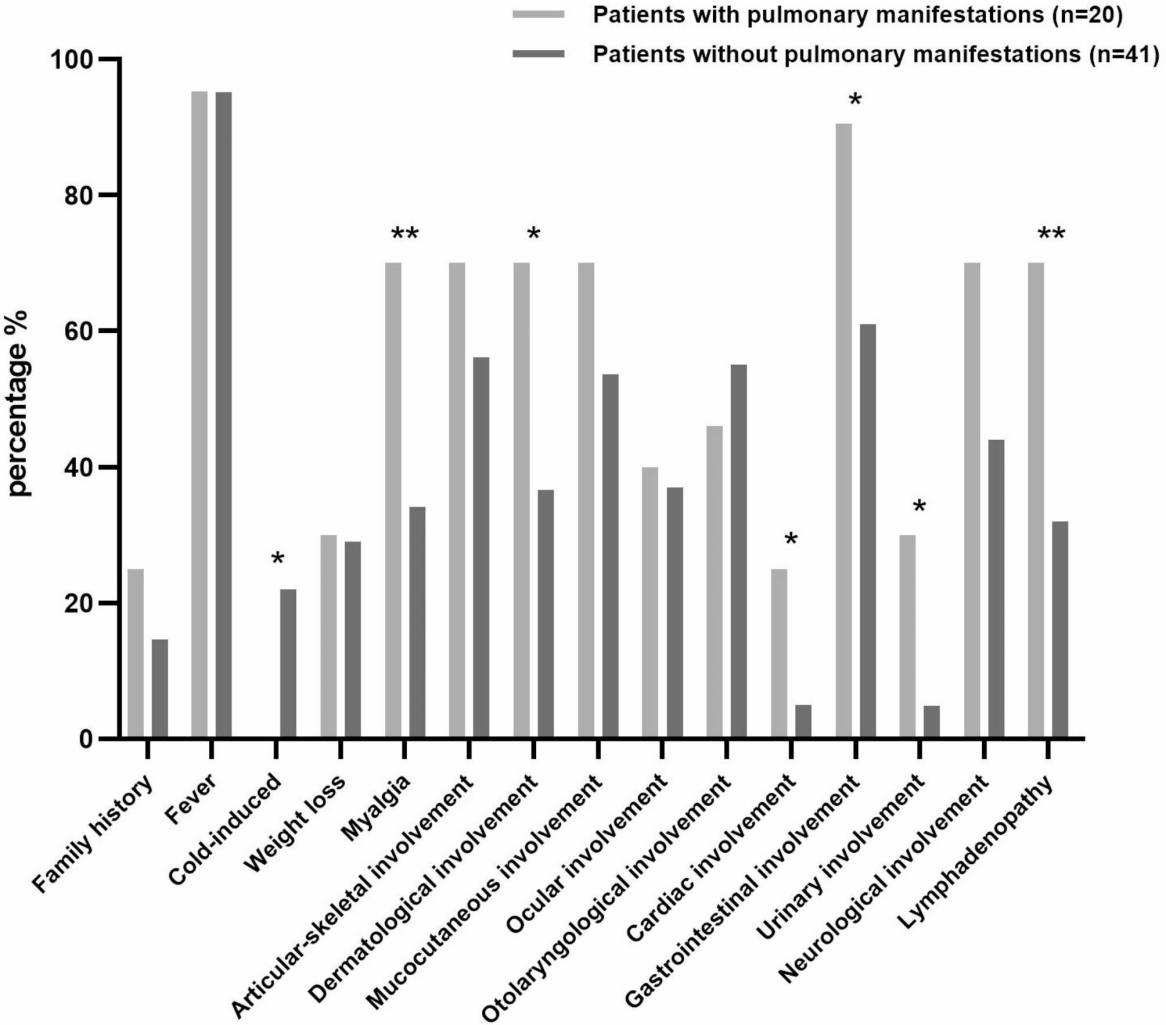
Comparison between the patients with pulmonary manifestations and those without

Upon grouping the 61 patients based on different organ involvements, we compared the occurrence of symptoms listed in Table 1 (data not shown). The results indicated that uSAID patients with pulmonary manifestations showed the most notable distinctions. Consequently, we compared the clinical data between patients with and without pulmonary manifestations. Pulmonary manifestations included cough, chest pain, pleural effusion/pleuritis, interstitial lung disease, pulmonary nodules confirmed by the physicians or imaging. Compared to patients without pulmonary manifestations, those with pulmonary involvement exhibited higher frequencies of myalgia (70% vs. 34%, *p* = 0.008), skin lesions (70% vs. 37%, *p* = 0.014), cardiac involvement (25% vs. 5%, *p* = 0.033), gastrointestinal involvement (90% vs. 61%, *p* = 0.02), urinary involvement (30% vs. 5%, *p* = 0.012), lymphadenopathy (70% vs. 32%, *p* = 0.005), neurological involvement including headaches (70% vs. 41%, *p* = 0.036), intellectual impairment (15% vs. 0, *p* = 0.032) and other rare manifestations (dizziness, epileptic seizure, intracranial calcification, relapsing meningitis and encephalatrophy) (30% vs. 5%, *p* = 0.012) (Fig. 2 and Supplement Table S2).

### Genotypes

**Fig. 3:**
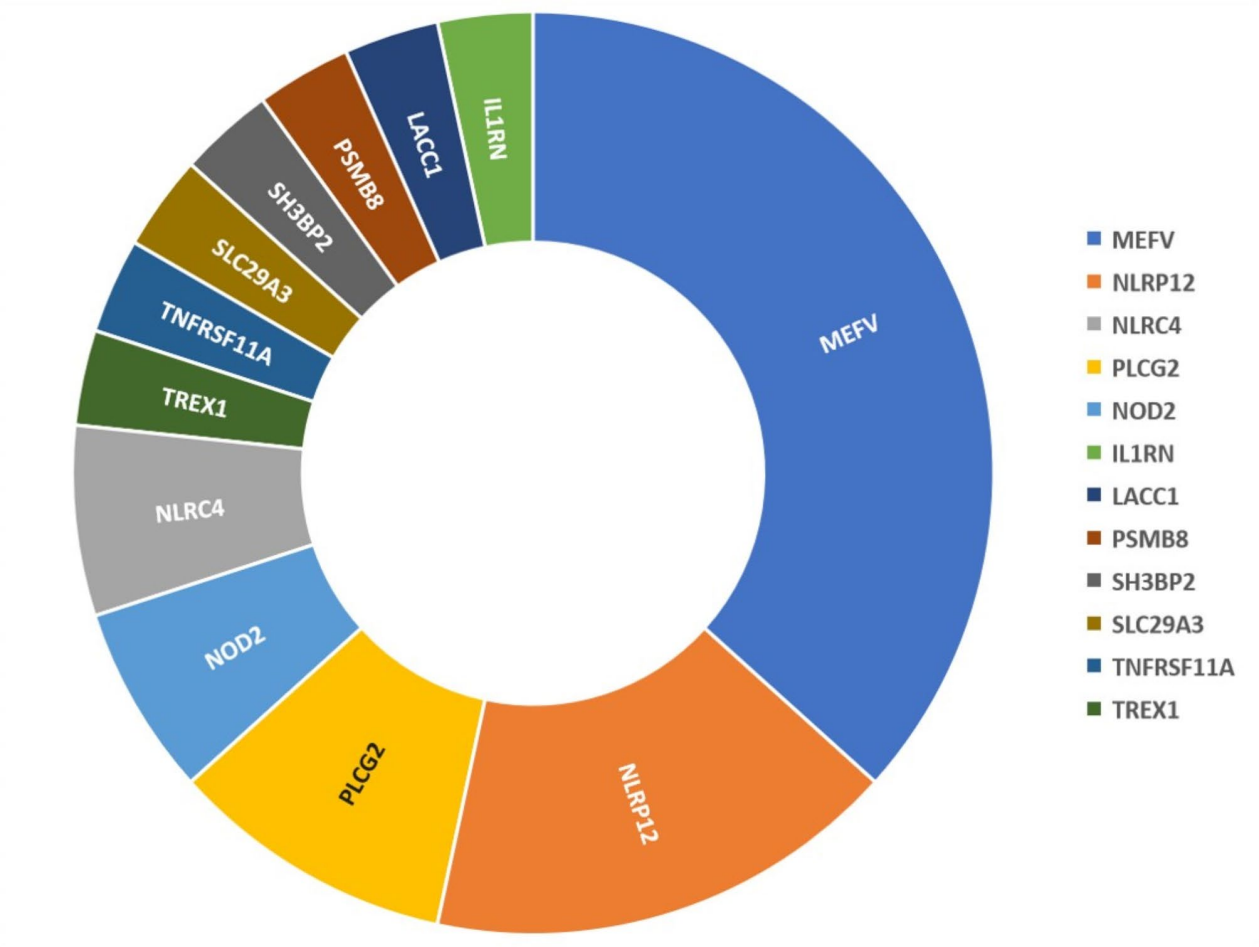
SAID-associated genes detected in uSAID patients

A total of 31 gene variants were identified among 24 patients (Fig. 3). Of these, 5 were common SNPs, and 10 were novel variants. The *MEFV* E148Q variant was the most prevalent among these patients. Patients carrying gene variants exhibited higher frequencies of cutaneous rash (64% vs. 36%, *p* = 0.032), erythema nodosum (36% vs. 8%, *p* = 0.01), pulmonary manifestations (48% vs. 22%, *p* = 0.035) and otolaryngological symptoms (64% vs. 33%, *p* = 0.018) compared to those without gene variants (data not shown).

### The subgroups of patients with uSAID

The genotypes and phenotypes of uSAID patients were categorized into three subgroups based on whether their phenotypes resembled any well-defined SAIDs, summarized in Table 2.

**Table 2:** Phenotypes and genotypes of uSAID patients

No.	Phenotypes	Clinical features	Genes	Mutations^#^
**Monogenic SAIDs-like Group: patients with monogenic SAIDs-like phenotypes**				
1–11	*NLRP3*-AID	Recurrent fever, arthralgia/arthritis, urticaria-like rash, ocular involvement, headache	*MEFV*	p.R408Q/P369S, p.E148Q
			*NOD2*	p.R541W
			*NLRP12*	p.F303fs^*^, *p.G39V*^$^
			*SHEBP2*	p.R263W^*^
			*TNFRSF11A*	p.P468S^*^
12–18	FMF	Recurrent fever, abdominal pain/diarrhea, arthralgia/arthritis		
19–21	TRAPS	Recurrent fever, myalgia, ocular involvement, arthralgia/arthritis, headache	*PLCG2*	p.H193Q
22–23	Interferonopathy	Recurrent fever, interstitial lung disease, pulmonary hypertension, soft tissue inflammation, cutaneous rash, myalgia	*PLCG2*	p.R448Q^*^
24	*NLRC4*-AID	Recurrent fever, pancytopenia, decreased NK cell viability, pericarditis, chest pain	*MEFV*	p.E148Q, p.P369S
**Polygenic SAIDs-like Group: patients with polygenic SAIDs-like phenotypes**				
25–27	PFAPA	Recurrent fever, pharyngitis, tonsillitis	*MEFV*	p.E148Q
28–31	IBD	Recurrent fever, abdominal pain/diarrhea, nausea/vomiting, gastrointestinal inflammation, erosion and ulcer showen by endoscopy	*NLRC4*	p.M775V^*^
			*NLRP12*	*p.F402L*
32–34	BS	Recurrent oral ulcer, conjunctivitis, pustular eruption	*PSMB8*	p.R163W
			*TREX1*	p.R15S^*^
35	Psoriasis	Recurrent fever, concomitant seborrheic dermatitis, scaly rash, acropachia		
36	CNO	Recurrent fever, pharyngitis, pustular eruption, inflammatory changes and bone marrow edema in the nasal bone, mandible and the left tibia		
**Atypical Group: patients with autoinflammatory features but not similar with any kind of known SAIDs**				
37–61	Unknown SAIDs	Recurrent fever, arthralgia/arthritis, erythema nodosa, headache, myalgia, pharyngitis, oral ulcer, ocular involvements, abdominal pain/diarrhea, lymphadenopathy, periarticular soft tissue inflammation, recurrent hypoimmunoglobulinia	*IFIH1*	p.D572G^*^
			*IL1RN*	p.Y138C^*^
			*LACC1*	p.L185F^*^
			*MEFV*	p.E148Q, p.P369S
			*NLRC4*	p.L70F
			*NLRP12*	*p.G39V*
			*NOD2*	*p.R471C*
			*PLCG2*	p.H193Q
			*SLC29A3*	p.A466G^*^

FMF: familial Mediterranean fever; *NLRP3*-AID: *NLRP3*-associated autoinflammatory disease; TRAPS: TNF receptor-associated periodic syndrome; *NLRC4*-AID: *NLRC4*-associated autoinflammatory diseases; NK cell: natural killer cell; PFAPA: periodic fever, aphthous stomatitis, pharyngitis and adenitis syndrome; BS: Behçet’s syndrome; IBD: inflammatory bowel disease; CNO: chronic nonbacterial osteomyelitis; PR: palindromic rheumatism; CVID: common variable immunodeficiency

^#^ All of the variants were heterozygous variants except one patient with *NLRP12* G903V variant in monogenic SAIDs-like group;

^$^ homozygous variant;

#### Monogenic SAID-like group

Among the 24 patients with monogenic SAID-like phenotypes, 11 presented with *NLRP3*-AID-like, 7 with FMF-like, 3 with TRAPS-like, 2 with interferonopathy-like and 1 with *NLRC4*-associated autoinflammatory disease (*NLRC4*-AID)-like symptoms. Although WES and Sanger sequencing targeted the suspected genes, molecular diagnoses couldn’t be confirmed, and some variants unrelated to the phenotypes were detected. According to established criteria [7], *MEFV* gene variants are not necessary for diagnosing FMF; however, 7 patients with FMF-like symptoms didn’t respond to colchicine and were thus diagnosed with uSAID instead of FMF.

#### Polygenic SAID-like group

In this cohort, 12 patients were classified into this group based on polygenic SAID-like phenotypes. Among them, 3 exhibited PFAPA-like symptoms, such as periodic fever, pharyngitis, and tonsillitis, but none experienced aphthous stomatitis or adenitis. Additionally, the frequency and episodes of fever were inconsistent with PFAPA, leading to a final diagnosis of uSAID rather than PFAPA. IBD was suspected in 4 patients due to clinical features such as recurrent fever, abdominal pain, diarrhea and weight loss, but endoscopy and pathology results did not support the diagnosis of IBD. Gene mutations related to IBD, such as *NOD2*, were not identified through NGS or Sanger sequencing. Three patients presented with recurrent oral ulcers, conjunctivitis and pustular eruption, resembling BS, but none fully met the criteria for BS. Furthermore, gene testing did not reveal variants in *TNFAIP3*, a gene related to haploinsufficiency of A20 (HA20), or other genes linked to BS [16]. One patient exhibited inflammatory changes and bone marrow edema in the nasal bone, mandible and left tibia, consistent with CNO, but also had recurrent fever, pharyngitis, and tracheochondritis which could not be explained by CNO. Another patient with a psoriasis-like phenotype had recurrent fever, seborrheic dermatitis, scaly rash, headache, arthralgia, and acropachy, none of which could be attributed solely to psoriasis.

#### Atypical group

The remaining 25 patients had a variety of clinical manifestations that did not resemble any well-defined SAIDs, leading to their classification into the atypical group. Common symptoms in this group included recurrent fever, arthralgia/arthritis, headache, erythema nodosa, myalgia, pharyngitis, ocular involvement, abdominal pain/diarrhea and lymphadenopathy. Among these patients, 5 developed recurrent erythema nodosum, scattering throughout the body particularly on the limbs, alongside recurrent fever. Two patients exhibited periodic arthritis and periarticular soft tissue inflammation similar to palindromic rheumatism (PR), but the urticaria-like rash in both and the recurrent fever in one could not be explained by PR. One patient was suspected of having common variable immunodeficiency disease (CVID) due to hypoimmunoglobulinemia with decreased immunoglobulin (Ig)A and IgG levels; however, this patient also experienced recurrent fever, headache, and tachycardia without evidence of infection.

### Treatment responses

Treatment responses are summarized in Table 3. 13% of patients (8/61) were lost to follow-up. Nonsteroidal anti-inflammatory drugs (NSAIDs) and corticosteroids were the most commonly used treatments (41/53, 77%). Although symptoms such as fever and arthralgia/arthritis were alleviated by NSAIDs and corticosteroids during episodes, the frequency of episodes did not decrease. Consequently, disease-modifying anti-rheumatic drugs (DMARDs) were often used (20/53, 38%) in combination with steroids, with good effectiveness in 30% patients (6/20), partial effectiveness in 45% (9/20), and ineffectiveness in 25% (5/20). Considering the effectiveness of colchicine in both well-defined SAIDs and uSAID patients especially for the serositis [17], treatments with colchicine were given to 13 patients, of whom 5 did not tolerate it well, and 3 did not follow the prescription due to spontaneous relief of symptoms. Among the 5 patients who received regular colchicine treatment, only one showed a good response, despite not having an FMF phenotype. Seven patients were treated with biological agents, including etanercept (*n* = 3), tofacitinib (*n* = 3), and baricitinib (*n* = 1). The effectiveness of biological agents was 100% for etanercept (complete remission) and 75% of Janus kinase inhibitors (25% complete remission, 50% partial remission). One patient with interferonopathy-like phenotype treated with tofacitinib died due to the progression of interstitial lung disease and lung infection.

**Table 3:** Treatment response of uSAID patients

Group	Monogenic SAID-like *n* = 24				Polygenic SAID-like *n* = 12				Atypical *n* = 25				Total *n* = 61			
Spontaneous improvement^*^	1				2				10				13			
Treatment response	GR	PR	NR		GR	PR	NR		GR	PR	NR		GR	PR	NR	
NSAIDs	-	9	2		1	1	1		1	4	-		2	14	3	
Corticosteroids	4	5	2		1	2	2		2	5	-		7	12	4	
DMARDs	3	3	3		1	3	1		2	3	1		6	9	5	
Colchicine	1	-	1		-	-	2		-	-	1		1	-	4	
Etanercept	1	-	-		1	-	-		1	-	-		3	-	-	
Tofacitinib	-	1	1		-	-	-		1	-	-		1	1	1	
Baricitinib	-	-	-		-	-	-		-	1	-		-	1	-	
Total	6	5	8		3	5	-		4	6	3		13	16	11	
Loss to follow-up	4				2				2				8			

GR: good response; PR: partial response; NR: no response; NSAIDs: nonsteroidal anti-inflammatory drugs; DMARDs: disease-modifying anti-rheumatic drugs;

^*^ Spontaneous improvements referred to the complete or partial self-relief of symptoms with no relation to the treatments

Overall, 43% (23/53) of patients showed good treatment responses, 36% (19/53) had partial responses, and 21% (11/53) had no response, with the highest rate of ineffectiveness observed in the monogenic SAID-like group (8/20, 40%). Additionally, during the follow-up of 53 patients, 25% (13/53) patients improved spontaneously with complete or partial recovery unrelated to therapy, including 1 from the monogenic SAID-like group (1/20, 5%), 2 from the polygenic SAID-like group (2/10, 20%) and 10 from the atypical group (10/23, 44%).

## Discussion

uSAID is a heterogeneous disorder characterized by sterile systemic inflammatory episodes not meet the criteria for well-defined SAIDs and lack a confirmed molecular diagnosis. To our knowledge, no studies have been conducted on Chinese adult patients with uSAID until now, making this study the first to describe the phenotypes, genotypes and treatment responses in this population.

There are currently no widely accepted criteria for diagnosing uSAID. In this study, the diagnostic criteria were primarily derived from previous studies [4, 6, 18–20], with modifications including follow-up and reanalysis based on the clinical experience of physicians at our center, which we believe helps avoid confounding diagnosis. Symptom diaries and responses to empirical therapies during follow-up are critical diagnostic tools. It is important to note that, as SAID is a rapidly expanding disease category with an increasing number of SAID-associated genes, patients diagnosed with uSAID today may receive a precise molecular diagnosis in the future if their variants are proven pathogenic. For instance, of the 70 presumed uSAID patients in our study, 4 were excluded during follow-up due to alterative diagnoses of RA and CNO based on symptom diaries or positive responses to empirical therapies for trial. Notably, 2 patients initially diagnosed with uSAID were later found to carry pathogenetic variants of the *PSTPIP1* or *ADA2* genes after 3 to 5 years of follow-up, leading to diagnoses of PAPA and DADA2, respectively [21, 22]. Therefore, we recommend continuous re-evaluation during follow-up and reanalysis of WES results to detect newly documented SAID-related gene variants for the accurate diagnosis of uSAID.

Our previous and other studies have shown that the clinical manifestations in adult patients with well-defined SAIDs are generally milder and more atypical compared to pediatric cases [23, 24]. Similarly, Ter Haar et al. observed a higher frequency of pericarditis, chest pain and intellectual impairments in child-onset patients compared to adult-onset uSAID patients [6]. In our study, we also found significant differences in the frequency of skin lesions, impaired vision and chest pain, with these symptoms being more prevalent in child-onset patients. However, further studies with large sample sizes are needed to verify these findings.

Intriguingly, we observed that patients with pulmonary manifestations had more distinctive phenotypes than those without. Pulmonary involvement is uncommon in well-defined SAIDs, except for chest pain due to sterile pleuritis in FMF and TRAPS, and interstitial lung disease in STING-associated vasculopathy with onset in infancy (SAVI), caused by the expression of STING protein in the alveolar epithelium [25]. Among the 20 patients with pulmonary manifestations in our cohort, half experienced pleuritis or chest pain, not limited in FMF-like and TRAPS-like phenotypes, and only 3 had interstitial lung disease with interferonopathy-like and atypical phenotypes. A case report also highlighted the uniqueness of pulmonary involvement in uSAID, describing a patient with BS-like phenotype whose systemic symptoms responded to anakinra immediately, but pulmonary symptoms only improved with tocilizumab [26]. This different response suggests that the pathogenesis of pulmonary symptoms in uSAID might differ from other symptoms and IL-6 might participate [26]. Additionally, a recent study found that 55% of patients with presumed uSAID had an elevated interferon signature, which correlated with more severe phenotypes [27]. Given that pulmonary involvement is a key feature of interferonopathy, the distinct characteristics of patients with pulmonary manifestations in our cohort might also be associated with IFN. However, one limitation of our study is the lack of interferon signature analysis for all uSAID patients, which could provide further insights.

In terms of the gene analysis, *MEFV* E148Q variant was the most common in our cohort, consistent with our previous findings in well-defined SAIDs [23]. Despite its high MAF of around 0.3 in East Asia, we did not filter this variant due to its potential pathogenic effects in FMF patients and its uncertain significance in the Infevers database [23]. Ter Haar et al. also noted that uSAID patients with genetic variants tended to have more distinctive phenotypes, similar to our findings, where these patients more frequently exhibited skin lesions, pulmonary manifestations and otolaryngological symptoms [6]. However, further research is needed to explore the correlations of genotypes and phenotypes in uSAID patients.

Consistent with other studies on well-defined SAIDs [28, 29], corticosteroids, NSAIDs and DMARDs were generally insufficient for treating our uSAID patients, while biological agents might offer a more effective therapeutic approach. However, there is limited evidence on the efficacy of biological agents for uSAID. Inspired by the treatments for well-defined SAIDs, Stephanie et al. and Suchika et al. found that anakinra was effective in both pediatric and adult uSAID patients who did not respond to corticosteroids or DMARDs, even in the absence of a firm molecular diagnosis [30, 31]. Additionally, a patient with a BS-like phenotype showed an excellent response to tocilizumab as we mentioned before [26], and Mark et al. reported the successful use of baricitinib in a patient with AOSD-like phenotype after failure of IL-1 and IL-6 inhibitors [26, 32]. Given that IL-1 inhibitors are not available in China, and the empirical trials of TNF-α inhibitors have been effective in Chinese adult patients with well-defined SAIDs [33, 34], we used TNFα blockade in three uSAID patients and observed good responses. Besides, Janus Kinase inhibitors also appeared to yield good or partial responses in uSAID patients.

Our results indicated that the prognosis of uSAID patients with monogenic SAID-like phenotypes seemed to be the worst, as they were treated with DMARDs and biological agents more frequently, yet many still did not respond to treatment and had the lowest self-remission rate among the three groups. Conversely, patients with atypical phenotypes showed better self-improvement with less use of DMARDs and biological agents. These findings suggest that the prognosis of uSAID patients may be related to their clinical phenotypes. Further studies are needed to determine whether this is due to random factors.

## Conclusion

This study represents the first cohort of Chinese adult patients with uSAID and provides a comprehensive summary of their phenotypes, genotypes and treatment responses categorized by clinical manifestations. Our findings suggest that the diagnosis of uSAID is an ongoing process that requires re-evaluation and follow-up. Moreover, greater clinical attention may be needed for uSAID patients with pulmonary involvement due to their distinctive phenotypes. Patients with atypical uSAID phenotypes might have a favorable self-remission rate and prognosis.

## Electronic Supplementary Material

Below is the link to the electronic supplementary material.

Supplementary Table S1

Supplementary Table S2

## Data Availability

Not applicable.

## Abbreviations

AOSD: Adult-onset Still’s disease
BS: Behçet’s syndrome
CNO: Chronic nonbacterial osteomyelitis
CVID: Common variable immunodeficiency disease
DADA2: Deficiency of adenosine deaminase 2
DMARDs: Disease-modifying anti-rheumatic drugs
FMF: Familial Mediterranean fever
HA20: Haploinsufficiency of A20
IBD: Inflammatory bowel disease
IL: Interlukin
MAF: Minor allele frequency
NGS: Next-generation sequencing
NLRC4-AID: NLRC4-associated autoinflammatory disease
NLRP3-AID: NLRP3-associated autoinflammatory disease
NSAIDs: Nonsteroidal anti-inflammatory drugs
PAPA: Pyogenic arthritis, pyoderma gangrenosum and acne
PFAPA: Periodic fever, aphthous stomatitis, pharyngitis and adenitis
PR: Palindromic rheumatism
SAIDs: Systemic autoinflammatory diseases
SAVI: STING-associated vasculopathy with onset in infancy
sJIA: Systemic juvenile idiopathic arthritis
SNP: Single nucleotide polymorphism
TNF: Tumor necrosis factor
TRAPS: TNF receptor-associated periodic syndrome
uSAID: Undifferentiated or undefined systemic autoinflammatory disease
WES: Whole-exome sequencing
