# Supplementary material for: Undifferentiated autoinflammatory disease in adults: a prospective study in 61 patients
Source: Orphanet J Rare Dis. 2025 Apr 8;20:165. doi: 10.1186/s13023-025-03685-5 (PMC11978157; doi:10.1186/s13023-025-03685-5)
Supplement: Supplementary file 1 — Supplementary Table S1 [file 13023_2025_3685_MOESM1_ESM.docx]

**Supplementary Table S1.** The SAID-associated genes included in the genetic analyses

| *ADA2* | *IL10RA* | *NOD2* | *RNASEH2C* |
| --- | --- | --- | --- |
| *ADAM17* | *IL10RB* | *OTULIN* | *SAMD9L* |
| *ADAR1* | *IL1RN* | *PLCG2* | *SAMHD1* |
| *ALPK1* | *IL36RN* | *POMP* | *SH3BP2* |
| *AP1S3* | *ISG15* | *PSMA3* | *SLC29A3* |
| *CARD14* | *LACC1* | *PSMB10* | *STING1* |
| *CDC42* | *LPIN2* | *PSMB4* | *TMEM173* |
| *CEBPE* | *MEFV* | *PSMB8* | *TNFAIP3* |
| *DNASE1* | *MVK* | *PSMB9* | *TNFRSF11A* |
| *DNASE1L3* | *NCSTN* | *PSMG2* | *TNFRSF1A* |
| *DNASE2* | *NLRC4* | *PSTPIP1* | *TRAP1* |
| *F12* | *NLRP1* | *RBCK1* | *TREX1* |
| *IFIH1* | *NLRP12* | *RIPK1* | *TRNT1* |
| *IKBKG* | *NLRP3* | *RNASEH2A* | *UBA1* |
| *IL10* | *NLRP7* | *RNASEH2B* | *WDR1* |
