# Supplementary material for: Undifferentiated autoinflammatory disease in adults: a prospective study in 61 patients
Source: Orphanet J Rare Dis. 2025 Apr 8;20:165. doi: 10.1186/s13023-025-03685-5 (PMC11978157; doi:10.1186/s13023-025-03685-5)
Supplement: Supplementary file 2 — Supplementary Table S2 [file 13023_2025_3685_MOESM2_ESM.docx]

**Supplementary Table S2.** Comparison of the clinical features in 61 uSAID patients with or without pulmonary manifestations

|  | Patients with pulmonary manifestations  n=20 | Patients without pulmonary manifestations  n=41 | *p-*values |
| --- | --- | --- | --- |
| Demographic data |  |  |  |
| Ratio of gender (M:F) | 9:11 | 27:14 | 0.12 |
| Age at disease onset, median, range, years old | 26 (0-63) | 26 (0-59) | 0.958 |
| Age at diagnosis, median, range, years old | 36 (17-64) | 33 (16-61) | 0.848 |
| Delayed diagnosis, median, range, years | 10 (1-30) | 8 (1-31) | 0.431 |
| Family history, n (%) | 5 (25) | 6 (15) | 0.479 |
| Clinical manifestations, n (%) |  |  |  |
| Constitutional symptoms | 20 (100) | 40 (98) | 1.000 |
| Weight loss | 6 (30) | 12 (29) | 0.953 |
| Fever | 19 (95) | 39 (95) | 1.000 |
| **Cold-induced** | **0 (0)** | **9 (22)** | **0.024** |
| Fatigue | 14 (70) | 27 (66) | 0.746 |
| **Myalgia** | **14 (70)** | **14 (34)** | **0.008** |
| Articular-skeletal involvement | 14 (70) | 23 (56) | 0.297 |
| Arthralgia/arthritis | 13 (65) | 23 (56) | 0.507 |
| **Dermatological involvement** | **14 (70)** | **15 (37)** | **0.014** |
| **Cutaneous rash** | **14 (70)** | **15 (37)** | **0.014** |
| Erythema nodosa | 4 (20) | 8 (20) | 1.000 |
| Mucocutaneous involvement | 8 (40) | 15 (37) | 0.796 |
| Oral ulcers | 4 (20) | 14 (34) | 0.255 |
| Dry mouth | 6 (30) | 7 (17) | 0.321 |
| Ocular involvement | 8 (40) | 15 (37) | 0.796 |
| Periorbital oedema | 1 (5) | 2 (5) | 1.000 |
| Dry eyes | 3 (15) | 4 (10) | 0.674 |
| Conjunctivitis | 3 (15) | 7 (17) | 1.000 |
| Impaired vision | 4 (20) | 2 (5) | 0.084 |
| Other ocular manifestations^$^ | 6 (30) | 4 (10) | 0.066 |
| Otolaryngological involvement | 11 (55) | 17 (41) | 0.319 |
| Sensorineural deafness | 3 (15) | 2 (5) | 0.319 |
| Tinnitus | 3 (15) | 2 (5) | 0.314 |
| Pharyngitis/ Tonsillitis | 7 (35) | 14 (34) | 0.947 |
| **Cardiac involvement** | **5 (25)** | **2 (5)** | **0.033** |
| Effusion/ Pericarditis | 3 (15) | 1 (2) | 0.099 |
| **Gastrointestinal involvement** | **18 (90)** | **25 (61)** | **0.02** |
| Abdominal pain/diarrhea | 6 (30) | 14 (34) | 0.746 |
| **Nausea/vomiting** | **10 (50)** | **8 (20)** | **0.014** |
| Splenomegaly | 7 (35) | 12 (29) | 0.402 |
| Other gastrointestinal manifestations^#^ | 10 (50) | 13 (32) | 0.166 |
| **Urinary involvement** | **6 (30)** | **2 (5)** | **0.012** |
| **Proteinuria** | **5 (25)** | **1 (2)** | **0.012** |
| Haematuria | 2 (10) | 1 (2) | 0.248 |
| Neurological involvement | 14 (70) | 18 (44) | 0.055 |
| **Headache** | **14 (70)** | **17 (41)** | **0.036** |
| **Intellectual impairments** | **3 (15)** | **0 (0)** | **0.032** |
| **Other neurological manifestations^£^** | **6 (30)** | **2 (5)** | **0.012** |
| **Lymphadenopathy** | **14 (70)** | **13 (32)** | **0.005** |

**In bold**: Symptoms with statistical significance (*p*-values<0.05) between patients with pulmonary manifestations and patients without such conditions;

^$^ Other ocular manifestations included keratitis, scleritis, uveitis and optic atrophy;

^#^ Other gastrointestinal manifestations included ulcers, hemorrhage, hepatomegaly, abnormal liver function and intestinal obstruction;

^£^ Other neurological manifestations included dizziness, epileptic seizure, intracranial calcification, relapsing meningitis and encephalatrophy.
